# Genetic dissection of canine hip dysplasia phenotypes and osteoarthritis reveals three novel loci

**DOI:** 10.1186/s12864-019-6422-6

**Published:** 2019-12-27

**Authors:** Lea Mikkola, Saila Holopainen, Tiina Pessa-Morikawa, Anu K. Lappalainen, Marjo K. Hytönen, Hannes Lohi, Antti Iivanainen

**Affiliations:** 1 Department of Veterinary Biosciences, University of Helsinki, P.O. Box 66 (Mustialankatu 1), FI-00014 Helsinki, Finland; 2 Department of Medical and Clinical Genetics, University of Helsinki, Helsinki, Finland; 3 Folkhälsan Research Center, Helsinki, Finland; 4 Department of Equine and Small Animal Medicine, University of Helsinki, Helsinki, Finland

**Keywords:** Hip dysplasia, Osteoarthritis, Dog, German shepherd, Genome-wide association study

## Abstract

**Background:**

Hip dysplasia and osteoarthritis continue to be prevalent problems in veterinary and human medicine. Canine hip dysplasia is particularly problematic as it massively affects several large-sized breeds and can cause a severe impairment of the quality of life. In Finland, the complex condition is categorized to five classes from normal to severe dysplasia, but the categorization includes several sub-traits: congruity of the joint, Norberg angle, subluxation degree of the joint, shape and depth of the acetabulum, and osteoarthritis. Hip dysplasia and osteoarthritis have been proposed to have separate genetic etiologies.

**Results:**

Using Fédération Cynologique Internationale -standardized ventrodorsal radiographs, German shepherds were rigorously phenotyped for osteoarthritis, and for joint incongruity by Norberg angle and femoral head center position in relation to dorsal acetabular edge. The affected dogs were categorized into mild, moderate and severe dysplastic phenotypes using official hip scores. Three different genome-wide significant loci were uncovered. The strongest candidate genes for hip joint incongruity were noggin (*NOG*), a bone and joint developmental gene on chromosome 9, and nanos C2HC-type zinc finger 1 (*NANOS1*), a regulator of matrix metalloproteinase 14 (*MMP14*) on chromosome 28. Osteoarthritis mapped to a long intergenic region on chromosome 1, between genes encoding for NADPH oxidase 3 (*NOX3*), an intriguing candidate for articular cartilage degradation, and AT-rich interactive domain 1B (*ARID1B*) that has been previously linked to joint laxity.

**Conclusions:**

Our findings highlight the complexity of canine hip dysplasia phenotypes. In particular, the results of this study point to the potential involvement of specific and partially distinct loci and genes or pathways in the development of incongruity, mild dysplasia, moderate-to-severe dysplasia and osteoarthritis of canine hip joints. Further studies should unravel the unique and common mechanisms for the various sub-traits.

## Background

Canine hip dysplasia (CHD) is a common multifactorial hereditary disorder that has perplexed dog owners, breeders as well as veterinarians and researchers for decades. A standardized system for CHD grading has been developed in the countries that belong to the Fédération Cynologique Internationale (FCI). The FCI score is divided into five categories alphabetically: A to E, where A is normal and E is severe CHD. In Finland, the FCI score is defined separately for both hip joints, hence the format is given as: left hip score / right hip score. The FCI score is determined from different ‘sub-traits’ of the hip: congruency of the joint, Norberg angle (NoA), subluxation degree of the joint, shape and depth of the acetabulum, and whether there are any visible signs of osteoarthritis (OA) in the joint or not. FCI has derived the grading rules, from which the Finnish Kennel Club (FKC) has defined their guidelines for radiographing and scoring hip dysplasia [1]. The above-mentioned sub-traits are not recorded for later use, only the hip score is stored in the FKC database.

As the FCI or any other combinatory score does not accurately correlate with the various CHD sub-traits, these have to be studied separately. NoA and femoral head center position in relation to dorsal acetabular edge (FHCDAE) reflect the incongruity of the hip joint, which impacts the development of CHD [2]. Hip joint laxity is a major contributor to the development of OA. However, OA is suggested to develop due to many simultaneous pathologies, which influence the central structures of the joint [3]. OA may have a distinct genetic background in relation to the other hip sub-traits [4–6].

The current consensus is that CHD is polygenic, and genetic contribution to the phenotype can vary from small to moderate [7–14]. Variation between breeds is evident from several studies [5, 7, 9, 10, 14–16]. Some breeds are more susceptible to the disorder than others. Labrador Retrievers [7, 10, 17], Bernese Mountain dogs [9], Golden Retrievers [18], and German Shepherds [4, 14, 16] have been under special interest in studies of CHD, and several genetic associations with different hip phenotypes have been reported in these breeds. Different breeding strategies have been proposed to improve hip health; estimated breeding values are generally considered the most efficient approach [4, 19–22]. Also, newer methods like genomic selection might bring a long awaited solution in the fight against this disorder [17, 23, 24].

To better understand the genetic etiology of CHD related phenotypes, we have carried out here a successful genome-wide association study (GWAS) in a cohort of over 750 well-phenotyped German Shepherds to map loci for CHD and related sub-traits. We report three loci with genome-wide significance and two suggestive loci for different traits with physiologically relevant candidate genes.

## Results

### The joint incongruity, measured as FHCDAE and NoA, map to chromosomes 9, 25 and 28

**Fig. 1:**
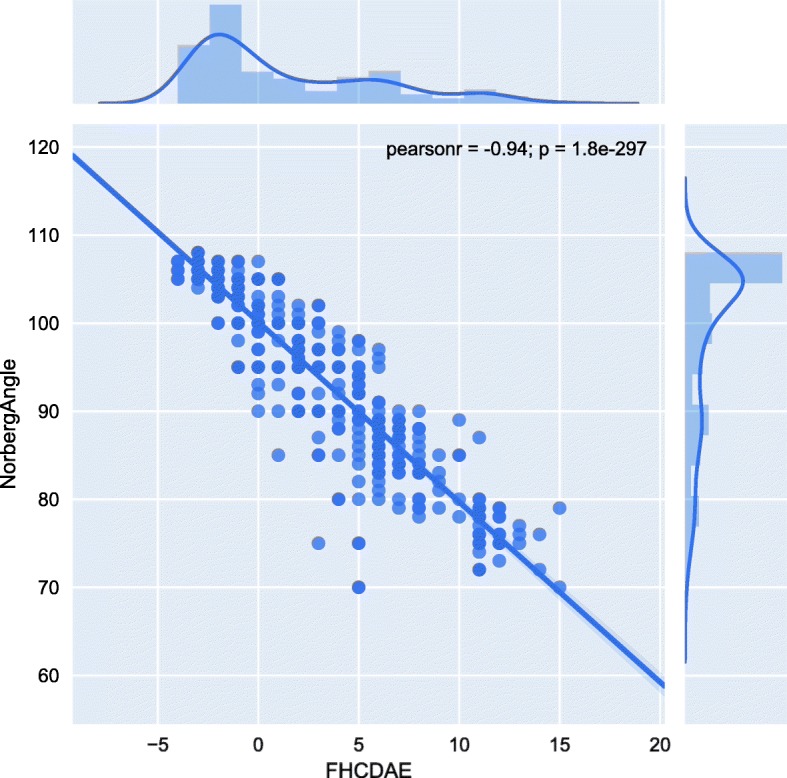
Correlation plot of NoA and FHCDAE**.** NoA is on the Y-axis and FHCDAE on the X-axis. Above the correlation plot is the distribution of FHCDAE measurements in the cohort. A respective distribution of the NoA measurements is on the right side of the correlation plot. Pearson’s *r* = − 0.94 and *P*-value = 1.8 × 10–297

Incongruity of the hip joint contributes to CHD. Therefore, we carried out two different association analyses on incongruity related traits, FHCDAE and NoA, which were assessed by two different veterinarians in our group. Both traits were measured for right and left hip, but we used only the worst measure in the analysis. NoA showed significant inter-observer variation in a linear regression model (*P* = 0.028, Additional file 1), which is consistent with earlier findings [25, 26]. Therefore, the evaluator was included as a covariate in the association analysis of NoA. For FHCDAE the inter-observer variation was non-significant. The association results for FHCDAE and NoA indicated overlapping loci, which is not surprising as these measurements were highly negatively correlated in the study cohort (Pearson’s r = − 0.94, Fig. 1). However, all the observed associations throughout the loci were stronger for FHCDAE than for NoA (Table 1).

**Table 1:** Top SNPs from the GWAS on FHCDAE and NoA

Trait	Chr	Locus	Alleles	N per SNP	SNP(s)	*P*-value from FASTA^(f)^, corrected with the inflation factor lambda (λ_FHCDAE_ = 1.000, λ_NoA_ = 1.003)
FHCDAE	9	31,477,907 31,387,114	G/A A/G	642 642	**BICF2G630834826** BICF2P742007	**1.57 × 10–6** 2.13 × 10–6
	28	29,111,565 29,122,985	A/C G/A	627 643	**BICF2P1046032** BICF2P895332	**1.62 × 10–6** 2.86 × 10–6
NoA	9	31,477,907 31,387,114 36,694,174	G/A A/G A/G	642 642 640	BICF2G630834826 BICF2P742007 BICF2G630837307	2.22 × 10–6 5.26 × 10–6 9.78 × 10–6
	25	10,301,514	A/C	640	BICF2G630468961	9.66 × 10–6

Single-nucleotide polymorphisms (SNPs) with probability values (*P*-values) < 1.0 × 10–5 are listed. SNPs and the corresponding *P*-values are in bold font, if the *P*-value passed the threshold for genome-wide significance (1.82 × 10–6) determined with the estimated number of independent tests from SimpleM (see methods). ^(f)^Family-based score test for association (FASTA). Chr = chromosome. N refers to the number dogs in analysis after exclusion of dogs by FASTA because they lack either the phenotype or a covariate. Covariates for NoA: age at radiographing, genetic cluster, genotyping batch, evaluator. Covariates for FHCDAE: age at radiographing, genetic cluster, and genotyping batch. See also Additional file 1

**Fig. 2:**
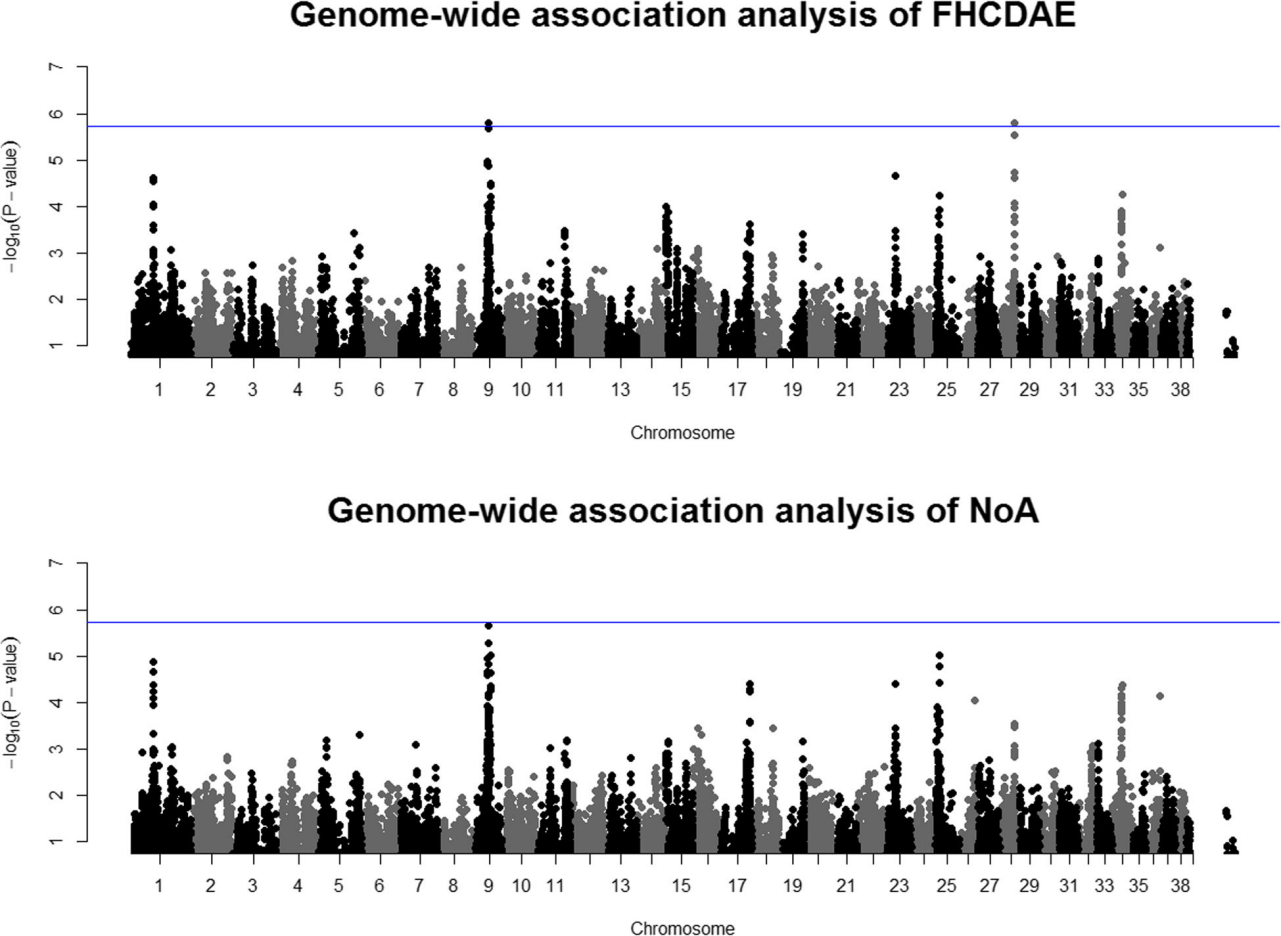
Manhattan plots for the analysis of hip joint incongruity traits FHCDAE and NoA. The upper Manhattan plot represents the results from the analysis of FHCDAE (*N* = 643). The blue line indicates the threshold for significance based on the number of independent tests. The lower plot represents the GWAS results of NoA (*N* = 642) with the blue line indicating the threshold for significance as in the upper plot

On chromosome 9, two SNPs demonstrated association with FHCDAE (Fig. 2). One of these SNPs passed the threshold for significance with independent tests (BICF2G630834826 with a *P*-value of 1.57 × 10–6, Table 1). BICF2G630834826 and BICF2P742007 are located ~ 22 kb downstream and ~ 67 kb upstream of *NOG* encoding noggin (Additional file 2), and they are in high linkage disequilibrium (LD) measured as the squared value (r^2^) of Pearson’s correlation coefficient between pairs of SNPs (r^2^ = 0.84, Additional file 3). These two SNPs also associated with NoA but the association was stronger for FHCDAE. The third SNP on chromosome 9, which was observed only for NoA (BICF2G630837307) and was not genome-wide significant, lies ~ 64 kb upstream of LIM homeobox 1 (*LHX1*) (Additional file 2).

Other loci with at least a suggestive association with the incongruity traits were on chromosomes 25 and 28 (Table 1, Fig. 2). On chromosome 25, BICF2G630468961 showing suggestive association with NoA was intronic to solute carrier family 7 member 1 (*SLC7A1*) (Additional file 2). On chromosome 28, SNPs BICF2P1046032 (in high LD with BICF2P895332; r^2^ = 0.96, Additional file 3) demonstrated significant association with FHCDAE (Table 1). These SNPs located between CDK2 associated cullin domain 1 (*CACUL1*) (~ 18 and 30 kb upstream, respectively) and nanos C2HC-type zinc finger 1 (*NANOS1*) (~ 163 and 174 kb upstream, respectively) (Additional file 2).

### OA maps to chromosome 1

**Fig. 3:**
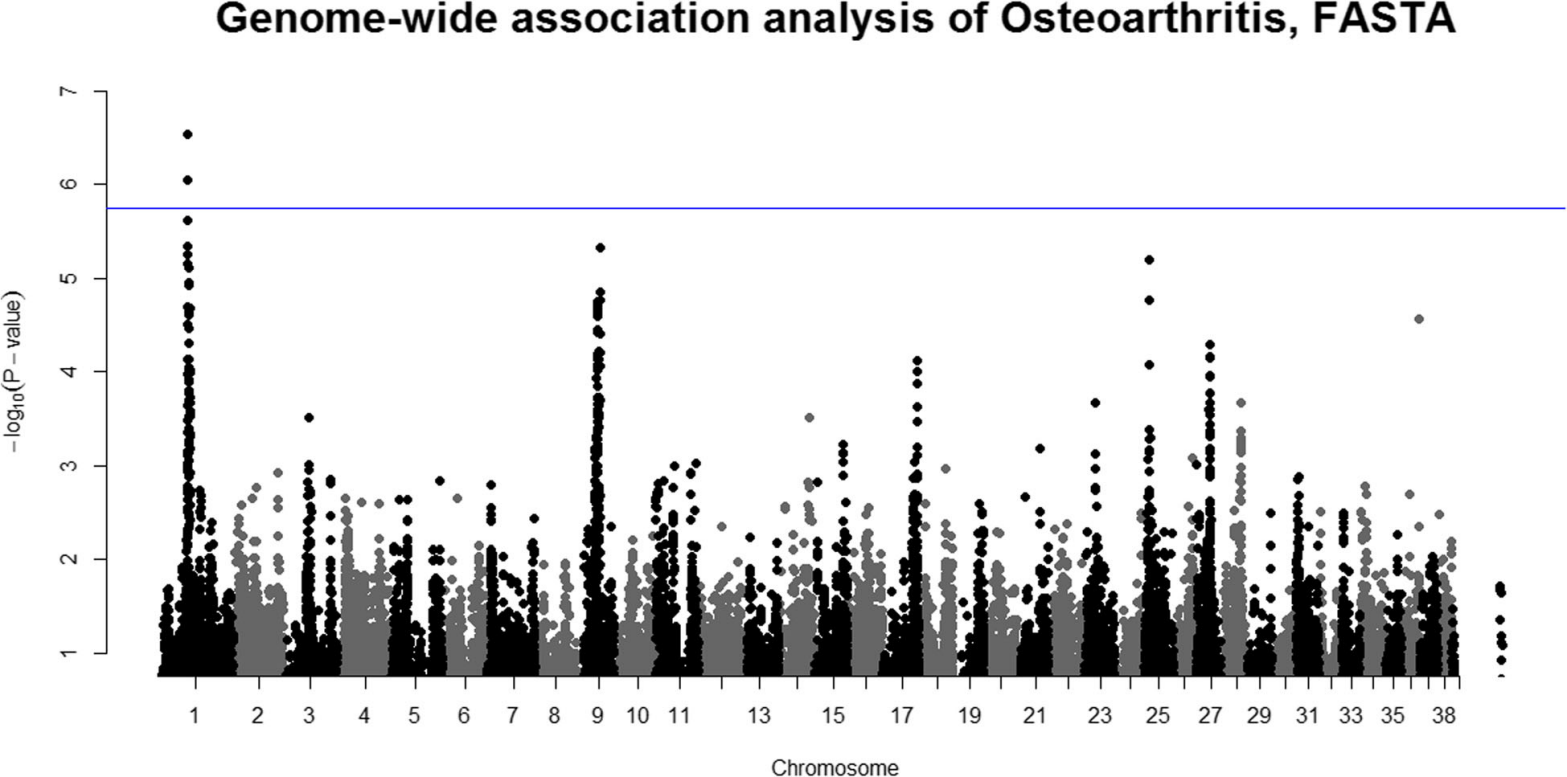
Manhattan plots for the binary trait: OA status. The Manhattan plot represents the lambda-corrected (lambda = 1.007) *P*-values from the FASTA analysis of osteoarthritis (*N* = 655), where the blue line shows the threshold for significance with independent tests

We studied OA as a separate disorder. Two veterinarians in our group evaluated the radiographs of individual dogs for evidence of OA (see methods). The dogs exhibited either no radiographic evidence of OA (controls) or had mild, moderate or severe signs of OA (cases). A case-control association analysis, where all controls (*N* = 492) were compared with all cases regardless of the severity of OA (*N* = 163), revealed a genome-wide significant locus on chromosome 1 (Fig. 3). The SNP with the strongest association (BICF2P468585) had a *P*-value of 2.86 × 10–7 (Table 2). The second-best SNP (BICF2P357728) reached a *P*-value of 8.93 × 10–7 (Table 2). Both SNPs passed the threshold for genome-wide significance based on the estimated number of independent tests determined with simpleM (1.82 × 10–6).

**Table 2:** Top SNPs from the GWAS on OA

Trait	Chr	Locus	Alleles	N per SNP	SNP(s)	*P*-value from FASTA, corrected with the inflation factor lambda (λ = 1.007)
OA status (binary; osteoarthritis present or not)	1	45,382,633 46,279,297 46,268,586 45,405,601 45,161,186 45,381,667 48,007,784 48,065,207	C/A A/G C/A G/A G/C G/A A/G A/G	655 650 654 652 655 655 655 655	**BICF2P468585** **BICF2P357728** BICF2P1037296 BICF2S23120955 BICF2S23248027 BICF2P392839 BICF2S23216908 BICF2S2305568	**2.86 × 10–7** **8.93 × 10–7** 2.39 × 10–6 4.48 × 10–6 5.62 × 10–6 7.05 × 10–6 7.81 × 10–6 7.81 × 10–6
	9	36,579,921	A/G	655	BICF2G630837240	4.69 × 10–6
	25	10,301,514	A/C	652	BICF2G630468961	6.22 × 10–6

SNPs with *P*-values < 1.0 × 10–5 are listed. The SNPs and their corresponding *P*-values are in bold font if the *P*-value passed the threshold for genome-wide significance (1.82 × 10–6) determined with the estimated number of independent tests from SimpleM (see methods). N refers to the number dogs in analysis after exclusion of dogs by FASTA because they lack either the phenotype or a covariate. Covariates for the OA status: age at radiographing, genetic cluster, genotyping batch and birth month. See also Additional file 1

The two genome-wide significant SNPs, as well as four out of the six SNPs showing suggestive association with OA on this chromosome, located between NADPH oxidase 3 (*NOX3*) (except BICF2S23248027, which lies within the ninth intron of *NOX3*) and AT-rich interaction domain 1B (*ARID1B*) (Table 2, Additional file 2). The top SNPs BICF2P468585 and BICF2P357728 were observed to be in high LD (r^2^ = 0.85, Additional file 3). Otherwise, moderate to perfect LD (r^2^ = 0.63–1.00) was observed between these six SNPs, even though the region they covered was over 1.1 Mb long (Additional file 3). Thus, we concluded that these SNPs probably represent just one locus that associates with the disorder. SNPs BICF2S23216908 and BICF2S2305568 (Table 2) are in perfect LD (r^2^ = 1.00, Additional file 3). Although they are ~ 1.7 Mb away from the other SNPs that associated with OA on this chromosome, we observed some LD between these two loci (r^2^ = 0.50–0.61, Additional file 3). BICF2S23216908 located within the first intron of Transmembrane protein 181 (*TMEM181*) and BICF2S2305568 within the first intron of Dynein light chain Tctex-type 1 (*DYNLT1*).

We also observed suggestive associations for chromosome 9 and 25 for OA. On chromosome 9, BICF2G630837240 locates ~ 101 kb downstream from *MRM1* encoding Mitochondrial RRNA Methyltransferase 1 and ~ 178 kb upstream from *LHX1* (Table 2, Additional file 2). BICF2G630468961 on chromosome 25 is located within the second intron of *SLC7A1* (Table 2 Additional file 2).

### Different genetic etiology of mild and moderate-to-severe CHD

To identify loci for CHD according to the FCI hip scores, we carried out three sets of case-control association analyses. In the first case-control analysis, the controls had a bilateral FCI hip score A and cases B/C, C/B or bilateral FCI score C or worse (N_cases_ = 339, N_controls_ = 354). In the second analysis, the same controls were used but cases had a bilateral FCI score of D or worse (N_cases_ = 166). In the third analysis we compared mild CHD dogs (B/C, C/B or bilateral FCI score C) with dogs that had moderate-to-severe (at least an FCI score D or worse for either hip) CHD (N_mild_ = 124, N_moderate-to-severe_ = 216). The summary of the results of these three comparisons are shown in Table 3.

**Table 3:** Top SNPs from the GWAS on different case-control analyses of the FCI hip score

Trait	Chr	Locus	Alleles	N per SNP	SNP(s)	*P*-value from FASTA, corrected with the inflation factor lambda (λ = 1.010–1.024)
1st case-control analysis (normal hips or mild-to-severe CHD; FCI scores)	1	45,382,633 45,161,186 46,279,297 46,268,586	C/A G/C A/G C/A	693 693 689 692	**BICF2P468585** **BICF2S23248027** BICF2P357728 BICF2P1037296	**7.03 × 10–7** **1.30 × 10–6** 2.52 × 10–6 9.51 × 10–6
2nd case-control analysis (normal hips or moderate-to-severe CHD; FCI scores)	9	36,837,067 36,886,621	G/A A/G	520 517	BICF2G630837405 TIGRP2P126345	4.96 × 10–6 7.75 × 10–6
3rd case-control analysis (mild CHD or moderate-to-severe CHD; FCI scores)	9	36,837,067 36,886,621	G/A A/G	340 338	BICF2G630837405 TIGRP2P126345	4.12 × 10–6 7.48 × 10–6

SNPs with *P*-values < 1.0 × 10–5 are listed. The SNPs and their corresponding *P*-values are in bold font if the P-value passed the threshold for genome-wide significance (1.82 × 10–6) determined with the estimated number of independent tests from SimpleM (see methods). N refers to the number dogs in analysis after exclusion of dogs by FASTA because they lack either the phenotype or a covariate. Covariates for the 1st analysis: age at radiographing and genotyping batch. Covariates for the 2nd analysis: age at radiographing, genetic cluster, genotyping batch and birth month. Covariates for the 3rd analysis: genetic cluster, genotyping batch and birth month. See also Additional file 1

**Fig. 4:**
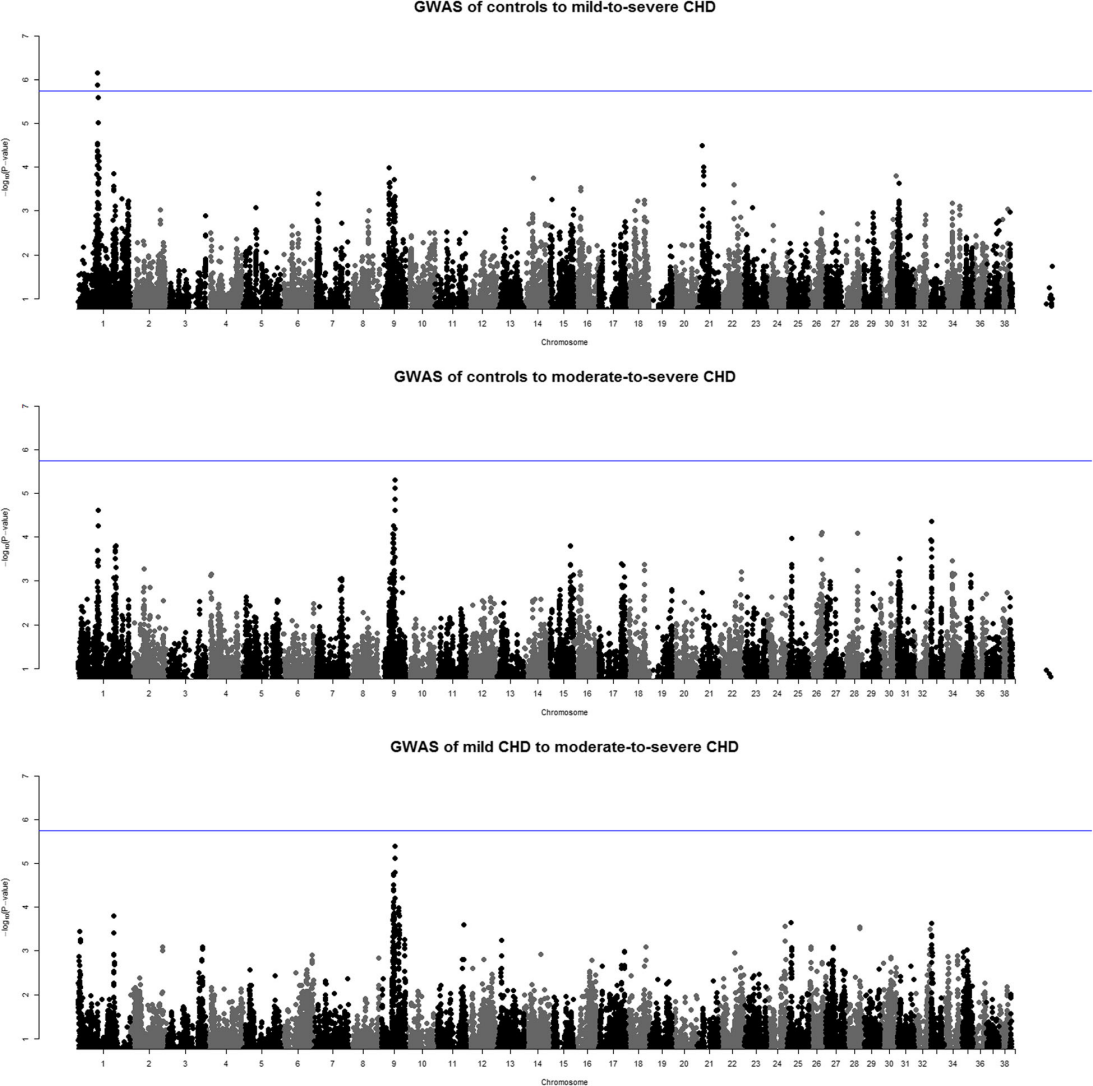
Manhattan plots for the case-control analyses of controls and mild to severe cases. The upmost Manhattan plot represents the case-control analysis, where controls were dogs with an FCI score A/A and cases were dogs with an FCI score B/C, C/B, or C or worse on both hips (*N* = 693). The second Manhattan plot represents the case-control analysis, where cases were dogs with an FCI score D or worse on both hips (*N* = 520), and the lowest Manhattan plot is the comparison between mild cases (B/C, C/B, C/C) to moderate-to-severe cases (D or worse on both hips) (*N* = 340). In each plot, the blue line shows the threshold for significance with independent tests

A genome-wide significant association was found on chromosome 1 for the first comparison with close to 700 dogs (Fig. 4 and Table 3). The SNPs with the strongest association (BICF2P468585 and BICF2S23248027) passed the threshold for significance with independent tests (Table 3). The identified locus between *NOX3* and *ARID1B* is the same we found for OA (Additional file 2). For the latter two case-control analyses with smaller number of dogs, none of the associations reached genome-wide significance. BICF2G630837405 on chromosome 9 lies within the eighth intron of apoptosis antagonizing transcription factor (*AATF*) and TIGRP2P126345 located ~ 8 kb downstream from the same gene. These two SNPs are in high LD (r^2^ = 0.97, Additional file 3).

A summary of the genome-wide significant loci across CHD-related traits described above are listed in Table 4. The frequencies of the effect and alternative alleles of the significantly associated SNPs in cases and controls (binary analyses) are in Additional file 4. Some SNPs were associated with more than one trait, as expected when the phenotypes are not independent from each other. The heritability (h^2^) estimates from the polygenic mixed model for the different traits varied from 36 to 64% (Additional file 5).

**Table 4:** Summary of the genome-wide significant SNPs for different CHD-related traits

Trait	Chr	Locus	Alleles	N per SNP	SNP(s)	*P*-value from FASTA, corrected with the inflation factor lambda
FHCDAE	9	31,477,907	G/A	642	BICF2G630834826	1.57 × 10–6
	28	29,111,565	A/C	627	BICF2P1046032	1.62 × 10–6
OA status (binary;osteoarthritis present or not)	1	45,382,633 46,279,297	C/A A/G	655 650	BICF2P468585 BICF2P357728	2.86 × 10–7 8.93 × 10–7
Case-control analysis (normal hips or mild-to-severe CHD; FCI scores)	1	45,382,633 45,161,186	C/A G/C	693 693	BICF2P468585 BICF2S23248027	7.03 × 10–7 1.30 × 10–6

The threshold for genome-wide significance was 1.82 × 10–6 (independent tests correction). The candidate genes in the immediate vicinity (± 200 kb) of the SNPs demonstrating genome-wide significance are in Table 5. All genes within ±1 Mb from the associated loci for all traits, for which *P*-value was < 1.0 × 10–5 are in Additional file 2

## Discussion

CHD is a complex skeletal disorder and one of the leading clinical concerns in veterinary medicine. CHD is categorically scored into five classes in screening programs of the FCI member countries but the phenotype manifests many sub-traits, which may eventually result in painful OA. The development of OA itself is a complex process, which involves alterations in many different tissues, including bone, cartilage, synovial membrane and ligaments [27]. Given the complexity of the disorder, it is not surprising that genetic discoveries have also remained scarce and breakthroughs require large and well-phenotyped study cohorts in each breed. We report here a remarkable progress by mapping three new loci on different chromosomes across key CHD traits in German Shepherds. The locus on chromosome 1 associated with OA and the FCI hip score, and the loci on chromosomes 9 and 28 associated with the trait FHCDAE, which measures hip joint incongruity (Table 4). In addition to the three loci with genome-wide significance, two suggestive loci on chromosomes 9 and 25 were uncovered for OA, NoA and different FCI hip score comparisons. Besides revealing novel loci, the study indicates that the locus on chromosome 1 associates with two binary traits: OA and the FCI hip score with relaxed case definition (B/C, C/B, or C or worse in both hips). Our study partially utilizes the study from Mikkola et al. (2019) [28] and as such cannot be regarded as an independent replication study.

The locus on chromosome 1 lies in a long intergenic region between *NOX3* and *ARID1B* (Table 5) Neither of the genes nor the intergenic region is known for functions that could explain their role in the development of CHD or OA. However, the likely significance of this locus for CHD is highlighted by the fact that our previously observed suggestive association [28] was strengthened by over ten times with a larger sample size. The association of the *NOX3-ARID1B* locus to OA was 2.5 times as strong as to the FCI hip score (as assessed by the ratio of the *P*-values). The latter is an aggregate phenotype and visible signs of OA (or the lack of them) are part of its evaluation. Therefore, it is not surprising to observe overlapping results.

**Table 5:** Candidate genes near SNPs showing genome-wide significant association with CHD-related phenotypes

Chr	Locus derived from the top SNP coordinates ± 200 kb	Gene(s)	OA	FHCDAE	FCI score A vs. FCI score C to E
1	44,961,186–46,479,297	*TIAM2, CLDN20, TFB1M,* ***NOX3***, ***ARID1B***	x		x
9	31,187,114–31,677,907	***NOG****, C9H17orf67, DGKE*		x	
28	28,911,565–29,322,985	*PRLHR,* ***CACUL1***, *NANOS1, EIF3A*		x	
**Total number of significant loci for the trait**			**1**	**2**	**1**

Bold font indicates the gene(s) closest to the associated SNP. The trait associations are marked with x

*NOX3* is a member of NADPH oxidases and an interesting candidate for articular cartilage degradation. NADPH oxidase participates in the generation of hydrogen peroxide, which is used by myeloperoxidase as a substrate to produce a highly reactive hypochlorous acid, and in some circumstances chlorine gas [29, 30]. These two reactive molecules oxidize the pyridinoline cross-links of articular cartilage and initiate its degradation [29, 30]. The SNP BICF2P468585 with the strongest association is ~ 196 kb upstream from *NOX3*, but BICF2S23248027 (also known as rs21911799) is located in the intron between *NOX3* exons 9 and 10 (Tables 4 and 5). Moreover, *NOX3* is mainly expressed in the inner ear and fetal tissues [31], thus, the role of *NOX3* in synovial tissue inflammation remains uncertain. Yet, among other protein-protein interactions, a STRING [32] database search (Additional file 6) suggested possible interplay between NOX3 and matrix metalloproteinases 2 and 9 – two matrix degrading enzymes implicated in CHD and OA [33–35]. We have previously discussed [28] that there is some evidence of the possible interplay between *NOX3* and *TRIO* (trio Rho guanine nucleotide exchange factor), another candidate gene for CHD [16]. The product of T-cell lymphoma invasion and metastasis 2 *(TIAM2)* further upstream (Table 5) modulates the activity of Rho-like proteins [36]. *ARID1B*, on the other hand, participates in transcriptional activation and repression through chromatin remodeling [37]. Interestingly, *ARID1B* is associated with joint laxity via a multisystemic Coffin-Siris syndrome (CSS); CSS is caused by *ARID1B* variants and 66% of the CSS patients exhibit joint laxity [38, 39].

Previous studies have suggested seven different loci for OA, none of them overlapping our loci. A multi-breed study by Zhou et al. (2010) [5] suggested two loci on canine chromosomes 17 and 37 for OA. Another quantitative trait locus (QTL) study in a crossbreed experiment reported putative QTLs on chromosomes 5, 18, 23 and 31 [6]. Chromosome 3 has also been suggested to harbor a QTL that regulates cranial and caudal acetabular osteophyte formation in Portuguese Water Dogs [40]. Discrepancy to our results may result from the genetic heterogeneity in different study populations, differences in analysis methods or phenotyping approaches in evaluating OA.

A locus in chromosome 9 near *NOG* associated with the incongruity trait FHCDAE (Tables 4 and 5). The association of the loci with NoA were weaker than with FHCDAE. This is not surprising as NoA suffers from high inter-observer variability [25, 26], which was also noted in our study. Similar bias was not seen for FHCDAE (Additional file 1). We previously found protective regulatory variants upstream *NOG*, and demonstrated the inverse correlation of their in vitro enhancer activity with healthy hips in German Shepherds [28]. The association of this locus with FHCDAE (as assessed by the ratio of the *P*-values) was ~ 24 times as strong as what we observed for the FCI hip score [28]. The putative contribution of *NOG* to FHCDAE remains elusive but may offer some leads to reduced joint congruity. Decreased noggin activity could possibly strengthen the acetabular bone via bone morphogenic protein (BMP) signaling and help the repair of microfractures and other damage caused by mechanical wear in growing dogs. Interestingly, delayed ossification of the femoral head has been associated with CHD in later life [41, 42]. *NOG* is a crucial gene for many developmental processes, such as neural tube fusion, joint formation and skeletal development [43, 44]. In humans, dominant *NOG* mutations cause some congenital disorders with abnormal joints [45], and knocking out murine *Nog* leads to a state where the mice lack most of the joints in the limbs [46]. On the other hand, overexpression of murine *Nog* results in osteopenia, bone fractures and decreased bone formation, when the function of osteoblasts becomes defective [47]. A recent study by Ghadakzadeh et al. (2018) [48] showed that knocking-down *Nog* in rats with small interfering RNA leads to down-regulation of *Nog* and increases both BMP-mediated differentiation of osteoblasts and the mineralization process of extracellular matrix.

The third locus with genome-wide significance involved also FHCDAE and resided on chromosome 28 (Tables 4 and 5). This region contains *CACUL1*, a cell-cycle associated gene [49], and *NANOS1* that upregulates *MMP14* a.k.a. membrane type 1-matrix metalloproteinase (*MT1-MMP*) thus promoting epithelial tumor cell invasion [50]. MT1-MMP is a powerful collagenolytic element [51, 52] and Miller et al. (2009) have demonstrated the role of MT1-MMP in human rheumatoid arthritis with synovial invasion via collagenolysis [53]. The possible role of the *NANOS1 – MMP14* interplay needs to be targeted in tissues relevant to CHD.

Intriguingly, chromosome 28 has been previously associated with NoA in two studies of which one included also German Shepherds [13, 54]. Although chromosome 28 did not associate with NoA in our study, the reported NoA locus is ~ 5.2 Mb upstream from our FHCDAE locus (Table 1). Because FHCDAE and NoA are strongly related traits (Pearsons’s r = − 0.94, Fig. 1), additional studies across breeds are warranted to find out whether the two loci on chromosome 28 are related or independent, and if they possess variants contributing to CHD.

We also observed some loci showing weaker associations with NoA and OA on chromosomes 9 and 25 (Tables 1 and 2), and with FCI hip score on chromosome 9 (Table 3). These loci included relevant candidate genes *LHX1*, *AATF* (both on chromosome 9) and *SLC7A1* (chromosome 25) (Additional file 2). *LHX1* could be a candidate for OA as it has been shown to be differentially methylated in OA [55] and is one of the most significantly up-regulated genes in this disorder [56]. SNPs near *LHX1* demonstrated also a suggestive association with CHD (quantified as the FCI hip score) in our previous study [28]. *AATF* is located close to *LHX1* but its role in CHD remains uncertain. Both *LHX1* and *AATF* have been associated with the levels of macrophage inflammatory protein 1b (MIP-1b) [57, 58]. MIP-1b is a cytokine increased in the synovial fluid in OA and may play a role in the ingression of monocytes into osteoarthritic joints [59]. The canine gene encoding MIP-1b (C-C motif for chemokine ligand 4*, CCL4*) is located on chromosome 9, ~ 795 kb away from TIGRP2P126345 and ~ 803 kb from *AATF* (Tables 1 and 3). SLC7A1 is a high affinity cationic amino acid transporter that belongs to the solute carrier family 7 [60]. It participates in the transportation of cationic amino acids arginine, lysine and ornithine across the plasma membrane [60]. L-arginine and its methylated forms could impact OA via the nitric oxide pathway [61].

Considering the clinical complexity of CHD, it is not surprising that we have successfully mapped several loci, which contain candidate genes that are involved in different biological pathways. Identification of these pathways is an important step in understanding the pathophysiology of CHD. Some of the genes in these networks may have no direct function on the disorder but have a circuitous effect through other genes [62]. As demonstrated here and previously by Sánchez-Molano et al. (2014) [7], the complexity and polygenicity of traits such as CHD required large sample sizes for significant associations. Sánchez-Molano et al. (2014) [7] had a cohort of 1500 Labrador Retrievers, and observed two genome-wide and multiple chromosome-wide significant QTLs explaining maximum 23% of the genetic variance in the analyzed traits. It is possible that larger cohorts might reveal additional loci with smaller effects.

Besides sample size, accurate and reliable phenotyping is another essential factor when studying complex traits. This is particularly important when the trait comprises of many interconnected sub-traits that explain only small parts of the total variation. As long as the assessment of CHD relies on the FCI scoring, it is crucial to have standardized high-quality radiographs and a minimal number of people assessing them to reduce inter-observer bias [26]. More reliable indices of joint laxity such as the distraction or laxity index [25], could facilitate the discovery of genetic findings by removing some confounding factors affecting NoA and FHCDAE, as some laxity remains undiscovered in the extended view radiographs.

## Conclusions

In conclusion, we have performed a successful association study with a large cohort of accurately and robustly phenotyped German Shepherds and describe three loci with genome-wide significance and two suggestive loci for CHD-related traits. The candidate genes include *NOX3* and *ARID1B* on chromosome 1, *NOG* on chromosome 9, and *NANOS1* on chromosome 28. Future studies will focus on ascertaining their role in CHD by resequencing the candidate region for putative risk variants.

## Methods

### Dogs

We acquired the data for our study from the Finnish Kennel Club. Before quality control we had a total of 775 samples of German Shepherds and of these 356 were controls, 322 were cases with both hip joints scored C or worse and 97 were of intermediate phenotypes with at least one hip joint scored as B. Majority of the dogs had either the same FCI score bilaterally or had maximum one score grade difference between the right and the left hip; three dogs had more than one grade difference (they had been scored A/C, C/A and B/D). The average age at radiographing was 1.55 years ranging from 1.01 to 5.83 years with a standard deviation of 0.63 years. 435 of the dogs were female and 340 were male. We collected at least one blood sample from all the dogs with ethylenediaminetetraacetic acid (EDTA) as an anticoagulant.

### Phenotypes

The FCI-standardized ventrodorsal extended hip radiographs were taken by different veterinarians, but the hip scoring was done by two specialized veterinarians at the FKC. Therefore, inter-observer bias was reduced in this data set [26]. All of the hip scores for these dogs are available in the FKC database [63]. We had at least a CHD score for all the dogs. We used the official FCI hip scores to divide the dogs into two different case-control groups: the first group with a relaxed case definition, where the cases had an FCI score B/C (left/right hip), C/B, or C/C or worse, and the second group with a stringent case definition, where the cases had an FCI score D or worse on both hips.

Two veterinarians in our group carefully evaluated the acquired radiographs for more specific hip phenotypes. These phenotypes were: findings suggestive on osteoarthritis (in four categories from 0 = no signs to 3 = severe signs), NoA (in degrees), and FHCDAE (in millimeters). The phenotyping process was carried out as follows: One veterinarian (evaluator 1 in the phenotype file doi: 10.6084/m9.figshare.10096595) assessed all the radiographs for the study cohort that was used in our previous study [28]. However, another veterinarian (evaluator 2 in the phenotype file (doi: 10.6084/m9.figshare.10096595) in our group evaluated the radiographs of the dogs that were genotyped during the current study. A small subset of randomly chosen radiographs, which the evaluator 1 had previously assessed were re-assessed by the evaluator 2 to check their consistency. In case there were any inconsistencies, the re-assessed phenotype was used in the analysis.

NoA varied between 70 and 108 degrees in our cohort (Table 6); the smaller the value is, the worse is the incongruity of the joint. Generally dogs with an FCI hip score A have NoA of 105 degrees or higher [64]. Significant inter-observer variation for NoA was seen in our data (*P* = 0.028, Additional file 1). We handled this in our GWAS by using the evaluator as a covariate. FHCDAE was measured as millimeters (mm) and in our data this trait ranged between − 4 and 15 mm (Table 6). The smaller the value is, the deeper the femoral head sits into the acetabulum in relation to the dorsal acetabular edge. OA was divided into four categories (the quantities for each category here are before quality control): no signs of arthritis (0, *N* = 498), some mild changes affiliated with OA (1, *N* = 57, minor osteophytes on the femoral neck and/or at the craniolateral acetabular edge), moderate changes (2, *N* = 74, larger osteophytes, also at the dorsal acetabular edge), or severe osteoarthritis (3, *N* = 33, massive osteophytes of the femoral neck and surrounding the acetabular edge). However, radiographs are relatively insensitive in detecting early osteoarthritic changes [65]. Therefore, the current study is unlikely to detect any associations with loci affecting exclusively the early stages of OA.

**Table 6:** Median, interquartile range and minimum and maximum values for the analyzed traits

Trait	Median	Interquartile range	25th percentile: 75th percentile	Min – Max
NoA (degrees)	102	15	90: 105	70–108
FHCDAE (mm)	0	7	−2: 5	−4 – 15
OA (category)	0	nr	nr	0–3

nr = not relevant

### DNA preparation and genotyping

The original EDTA preserved blood samples for this study are stored at the Dog DNA bank at the University of Helsinki. The DNA was extracted from these samples with a Chemagic Magnetic Separation Module I with a standard protocol by Chemagen (Chemagen Biopolymer-Technologie AG, Baeswieler, Germany). Thereafter the DNA samples were genotyped at Geneseek (Lincoln, NE, US) with a high density 173 K canine SNP array from Illumina (San Diego, CA, US). Genotyping of the samples was done in multiple batches.

### Population structure

We used information from a genomic relationship matrix built from the SNP data to divide our highly stratified German Shepherd population into three subpopulations (Additional file 7). For the clustering we used an R [66] package “mclust” [67] that utilizes covariance parametrization. The selection of appropriate number of clusters was executed with Bayesian information criterion. We then created a covariate vector from the clustering data where every individual belonged to one of the clusters. This way we could use the clustering effect in our model to account for any differences in disease association between the genetic clusters.

### Quality control (QC)

We used PLINK [68] to merge the original three genotype sets from different genotyping batches. A preliminary QC was done on all of the genotyping batches before merging, with the following thresholds: call rate per sample 0.10, call rate per SNP 0.05, minor allele frequency 0.05, *P*-value cut-off for deviation from Hardy-Weinberg equilibrium (HWE) 0.00001 (from controls only). After these quality controls and data merging a total of 100,435 SNPs and 775 samples were transferred from PLINK to R. The final QC was done in R with GenABEL [69], and the thresholds were: minor allele frequency = 0.05, per sample call rate = 0.85 and per SNP call rate = 0.95, and again a P-value cut-off level < 0.00001 to test for deviations from HWE. After the final QC we had 89,251 autosomal SNPs and 769 samples to use in our association analysis. However, the final number of dogs per analysis varied between 338 and 693 as FASTA dropped individual dogs from analyses if they missed a phenotype or a covariate. CanFam3.1 was used as the position map for our SNPs [70]. After the GWAS the genotype call quality of the top SNPs was checked to exclude associations due to calling errors.

### Genome-wide association analysis (GWAS)

We performed a GWAS by using polygenic mixed models in GenABEL, with functions “polygenic” and “mmscore” (FASTA: Score test for association in related people) [71]. The appropriate covariates were estimated with fitting linear regression models with the R function “lm” from the stats-package [72] for all non-binary traits. The binary traits were analyzed with fitting generalized linear models with the R function “glm” [73]. The following covariates were tested: sex, age at radiographing, genetic cluster of the dog, genotyping batch, birth month, and evaluator, in other words the veterinarian who evaluated the radiographs (tested for traits NoA, FHCDAE and OA). The appropriate covariates which had a significant effect (P-value < 0.05) for each dependent trait are in Table 7 (See also Additional file 1). The inflation factor lambda for the various models are indicated in the Tables 1-3. The corresponding Q-Q plots are in the Additional file 8.

**Table 7:** Covariates for different traits

Trait	Covariates chosen with lm or glm function in R
OA status (binary; osteoarthritis present or not)	Age at radiographing, genotyping batch, genetic cluster and birth month
FHCDAE	Age at radiographing, genetic cluster, genotyping batch
NoA	Age at radiographing, genetic cluster, genotyping batch, evaluator
Cases vs. controls (FCI score categories A vs. C-E)	Age at radiographing and genotyping batch
Cases vs. controls (FCI score categories A vs D-E)	Age at radiographing, genetic cluster, genotyping batch and birth month
Cases vs. controls (FCI score categories C vs D-E)	Genetic cluster, genotyping batch and birth month

The r^2^ values for the top SNPs were estimated in R with “r2fast” function [74] from GenABEL-package.

Bonferroni correction can be seen as a too stringent method to correct for multiple testing as it expects independency between the tests, which is untrue in many association studies because of LD between markers [75]. This is especially important to note in canine studies, as the structure of the canine genome is unique with strong LD due to the history of intensive selection [13]. Therefore, we used the number of independent tests to determine the threshold for significance. We estimated the effective number of independent tests to be 27,456 using simpleM, which uses dimension reduction models for filtering the correlations between the analyzed SNPs [76]. Based on this, the threshold for significance 1.82 × 10–6 (0.05/27456) is applied for *P*-values in this study.

## Supplementary information

**Additional file 1.** Summaries of glm and lm models

**Additional file 2.** List of genes within +/− 1 Mb from the associated loci

**Additional file 3.** Pairwise LD (r^2^) for the top SNPs by chromosome

**Additional file 4.** Allele frequencies for the significant SNPs from the case-control analyses

**Additional file 5.** Heritability (h^2^) estimates from the polygenic model (GenABEL) for all of the traits

**Additional file 6 **Results from a STRING database search for the candidate gene *NOX3*

**Additional file 7.** Classification plot of the population structure from R-package “mclust”. The population is divided into three subpopulations, where the green triangles represent individual dogs of one subpopulation and the red squares and the blue dots represent the individual dogs of the two other subpopulations

**Additional file 8.** Q-Q plots of the various association analyses corresponding to Tables 1-4

## Data Availability

The datasets generated and analyzed in the current study are available at FIGSHARE, doi: 10.6084/m9.figshare.10096595. The datasets were anonymized to protect the owners of the animals.

## Abbreviations

AATF: Apoptosis antagonizing transcription factor
ARID1B: AT-rich interactive domain 1B
BMP: Bone morphogenetic protein
CACUL1: CDK2 associated cullin domain 1
CCL4: C-C motif chemokine ligand 4
CHD: Canine hip dysplasia
Chr: Chromosome
EDTA: Ethylenediaminetetraacetic acid
FASTA: Family-based score test for association
FCI: Fédération cynologique internationale
FHCDAE: Femoral head center position in relation to dorsal acetabular edge
FKC: Finnish kennel club
GWAS: Genome-wide association study
h^2^: Heritability (narrow sense)
HWE: Hardy-Weinberg equilibrium
LD: Linkage disequilibrium
LHX1: LIM homeobox 1
MIP-1b: Macrophage inflammatory protein 1b
MMP14: Matrix metalloproteinase-14
MT1-MMP: Membrane type 1-matrix metalloproteinase
NANOS1: Nanos C2HC-type zinc finger 1
NoA: Norberg angle
NOG: Noggin
NOX3: NADPH oxidase 3
OA: Osteoarthritis
*P*-value: Probability value
QC: Quality control
QTL: Quantitative trait locus
r^2^: Square of pearson’s correlation coefficient r
SLC7A1: Solute carrier family 7 member 1
SNP: Single-nucleotide polymorphism

## References

[1] Brass W (1993). Hüftgelenkdysplasie und Ellbogenkrankkung im Visier der Fédération Cynologique Internationale. Kleintierpraxis.

[2] Skurková L, Hluchý M, Lacková M, Mihalová M, Ledecký V (2010). Relation of the norberg angle and position of the femoral head Centre to the dorsal acetabular edge in evaluation of canine hip dysplasia. Vet Comp Orthop Traumatol.

[3] Allen MJ (2016). What’s new in Orthopaedic basic science. J Bone Jt Surg.

[4] Wilson BJ, Nicholas FW, James JW, Wade CM, Raadsma HW, Thomson PC (2013). Genetic correlations among canine hip dysplasia radiographic traits in a cohort of Australian German shepherd dogs, and implications for the design of a more effective genetic control program. PLoS One.

[5] Zhou Z, Sheng X, Zhang Z, Zhao K, Zhu L, Guo G (2010). Differential genetic regulation of canine hip dysplasia and osteoarthritis. PLoS One.

[6] Mateescu RG, Burton-Wurster NI, Tsai K, Phavaphutanon J, Zhang Z, Murphy KE (2008). Identification of quantitative trait loci for osteoarthritis of hip joints in dogs. Am J Vet Res.

[7] Sánchez-Molano E, Woolliams JA, Pong-Wong R, Clements DN, Blott SC, Wiener P (2014). Quantitative trait loci mapping for canine hip dysplasia and its related traits in UK Labrador retrievers. BMC Genomics.

[8] Janutta V, Hamann H, Distl O (2006). Complex segregation analysis of canine hip dysplasia in German shepherd dogs. J Hered.

[9] Pfahler S, Distl O (2012). Identification of quantitative trait loci (QTL) for canine hip dysplasia and canine elbow dysplasia in Bernese mountain dogs. PLoS One.

[10] Lavrijsen ICM, Leegwater PAJ, Martin AJ, Harris SJ, Tryfonidou MA, Heuven HCM (2014). Genome wide analysis indicates genes for basement membrane and cartilage matrix proteins as candidates for hip dysplasia in Labrador retrievers. PLoS One.

[11] Todhunter RJ, Bliss SP, Casella G, Wu R, Lust G, Burton-Wurster NI (2003). Genetic structure of susceptibility traits for hip dysplasia and microsatellite Informativeness of an outcrossed canine pedigree. J Hered..

[12] Mäki K, Janss LLG, Groen AF, Liinamo A-E, Ojala M (2004). An indication of major genes affecting hip and elbow dysplasia in four Finnish dog populations. Hered (Edinb).

[13] Hayward JJ, Castelhano MG, Oliveira KC, Corey E, Balkman C, Baxter TL (2016). Complex disease and phenotype mapping in the domestic dog. Nat Commun.

[14] Fels L, Distl O (2014). Identification and validation of quantitative trait loci (QTL) for canine hip dysplasia (CHD) in German shepherd dogs. PLoS One.

[15] Bartolomé N, Segarra S, Artieda M, Francino O, Sánchez E, Szczypiorska M (2015). A genetic predictive model for canine hip dysplasia: integration of genome wide association study (GWAS) and candidate gene approaches. PLoS One.

[16] Fels L, Marschall Y, Philipp U, Distl O (2014). Multiple loci associated with canine hip dysplasia (CHD) in German shepherd dogs. Mamm Genome.

[17] Sánchez-Molano E, Woolliams JA, Blott SC, Wiener P (2014). Assessing the impact of genomic selection against hip dysplasia in the Labrador retriever dog. J Anim Breed Genet.

[18] Smith GK, Mayhew PD, Kapatkin AS, McKelvie PJ, Shofer FS, Gregor TP (2001). Evaluation of risk factors for degenerative joint disease associated with hip dysplasia in German shepherd dogs, Golden retrievers, Labrador retrievers, and Rottweilers. J Am Vet Med Assoc.

[19] Hou Y, Wang Y, Lu X, Zhang X, Zhao Q, Todhunter RJ (2013). Monitoring hip and elbow dysplasia achieved modest genetic improvement of 74 dog breeds over 40 years in USA. PLoS One.

[20] Lewis TW, Blott SC, Woolliams JA (2013). Comparative analyses of genetic trends and prospects for selection against hip and elbow dysplasia in 15 UK dog breeds. BMC Genet.

[21] Edwards SM, Woolliams JA, Hickey JM, Blott SC, Clements DN, Sánchez-Molano E (2018). Joint genomic prediction of canine hip dysplasia in UK and US Labrador retrievers. Front Genet.

[22] Oberbauer AM, Keller GG, Famula TR (2017). Long-term genetic selection reduced prevalence of hip and elbow dysplasia in 60 dog breeds. PLoS One.

[23] Sánchez-Molano E, Pong-Wong R, Clements DN, Blott SC, Wiener P, Woolliams JA (2015). Genomic prediction of traits related to canine hip dysplasia. Front Genet.

[24] Guo G, Zhou Z, Wang Y, Zhao K, Zhu L, Lust G (2011). Canine hip dysplasia is predictable by genotyping. Osteoarthr Cartil.

[25] Broeckx BJG, Vezzoni A, Bogaerts E, Bertal M, Deforce D, Peelman L (2018). Comparison of three methods to quantify laxity in the canine hip joint. Vet Comp Orthop Traumatol.

[26] Verhoeven GE, Coopman F, Duchateau L, Bosmans T, Van Ryssen B, Van Bree H (2009). Interobserver agreement of the assessability of standard ventrodorsal hip-extended radiographs and its effect on agreement in the diagnosis of canine hip dysplasia and routine FCI scoring. Vet Radiol Ultrasound.

[27] Loeser RF, Goldring SR, Scanzello CR, Goldring MB (2012). Osteoarthritis: a disease of the joint as an organ. Arthritis Rheum.

[28] Mikkola LI, Holopainen S, Lappalainen AK, Pessa-Morikawa T, Augustine TJP, Arumilli M (2019). Novel protective and risk loci in hip dysplasia in German shepherds. PLoS Genet.

[29] Daumer KM, Khan AU, Steinbeck MJ (2000). Chlorination of pyridinium compounds. Possible role of hypochlorite, N-chloramines, and chlorine in the oxidation of pyridinoline cross-links of articular cartilage collagen type II during acute inflammation. J Biol Chem.

[30] Steinbeck MJ, Nesti LJ, Sharkey PF, Parvizi J (2007). Myeloperoxidase and chlorinated peptides in osteoarthritis: potential biomarkers of the disease. J Orthop Res.

[31] Breitenbach M, Rinnerthaler M, Weber M, Breitenbach-Koller H, Karl T, Cullen P (2018). The defense and signaling role of NADPH oxidases in eukaryotic cells. Wien Med Wochenschr.

[32] Szklarczyk D, Franceschini A, Wyder S, Forslund K, Heller D, Huerta-Cepas J (2015). STRING v10: protein–protein interaction networks, integrated over the tree of life. Nucleic Acids Res.

[33] Volk SW, Kapatkin AS, Haskins ME, Walton RM, D’Angelo M (2003). Gelatinase activity in synovial fluid and synovium obtained from healthy and osteoarthritic joints of dogs. Am J Vet Res.

[34] Fujiki M, Shineha J, Yamanokuchi K, Misumi K, Sakamoto H (2007). Effects of treatment with polysulfated glycosaminoglycan on serum cartilage oligomeric matrix protein and C-reactive protein concentrations, serum matrix metalloproteinase-2 and -9 activities, and lameness in dogs with osteoarthritis. Am J Vet Res.

[35] Zeng GQ, Chen AB, Li W, Song JH, Gao CY (2015). High MMP-1, MMP-2, and MMP-9 protein levels in osteoarthritis. Genet Mol Res.

[36] Chiu CY, Leng S, Martin KA, Kim E, Gorman S, Duhl DM (1999). Cloning and characterization of T-cell lymphoma invasion and metastasis 2 (TIAM2), a novel guanine nucleotide exchange factor related to TIAM1. Genomics.

[37] ARID1B. Uniprot. https://www.uniprot.org/uniprot/Q8NFD5#function. Accessed 23 Nov 2018.

[38] Schrier Vergano S, Santen G, Wieczorek D, Wollnik B, Matsumoto N, Deardorff MA, Coffin-Siris Syndrome (1993). GeneReviews®.

[39] Santen GWE, Clayton-Smith J (2014). The *ARID1B* phenotype: what we have learned so far. Am J Med Genet Part C Semin Med Genet.

[40] Chase K, Lawler DF, Carrier DR, Lark KG (2005). Genetic regulation of osteoarthritis: a QTL regulating cranial and caudal acetabular osteophyte formation in the hip joint of the dog (Canis familiaris). Am J Med Genet A.

[41] Todhunter RJ, Zachos TA, Gilbert RO, Erb HN, Williams AJ, Burton-Wurster N (1997). Onset of epiphyseal mineralization and growth plate closure in radiographically normal and dysplastic Labrador retrievers. J Am Vet Med Assoc.

[42] Madsen JS, Reimann I, Svalastoga E (1991). Delayed ossification of the femoral head in dogs with hip dysplasia. J Small Anim Pract.

[43] McMahon JA, Takada S, Zimmerman LB, Fan CM, Harland RM, McMahon AP (1998). Noggin-mediated antagonism of BMP signaling is required for growth and patterning of the neural tube and somite. Genes Dev.

[44] Brunet LJ, McMahon JA, McMahon AP, Harland RM (1998). Noggin, cartilage morphogenesis, and joint formation in the mammalian skeleton. Sci.

[45] Potti TA, Petty EM, Lesperance MM (2011). A comprehensive review of reported heritable noggin-associated syndromes and proposed clinical utility of one broadly inclusive diagnostic term: NOG-related-symphalangism spectrum disorder (NOG-SSD). Hum Mutat.

[46] Tylzanowski P, Mebis L, Luyte FP (2006). The noggin null mouse phenotype is strain dependent and haploinsufficieny leads to skeletal defects. Dev Dyn.

[47] Devlin RD, Du Z, Pereira RC, Kimble RB, Economides AN, Jorgetti V (2003). Skeletal overexpression of noggin results in osteopenia and reduced bone formation. Endocrinol.

[48] Ghadakzadeh S, Hamdy RC, Tabrizian M (2017). Efficient in vitro delivery of noggin siRNA enhances osteoblastogenesis. Heliyon.

[49] Kong Y, Nan K, Yin Y (2009). Identification and characterization of CAC1 as a novel CDK2-associated cullin. Cell Cycle.

[50] Bonnomet A, Polette M, Strumane K, Gilles C, Dalstein V, Kileztky C (2008). The E-cadherin-repressed hNanos1 gene induces tumor cell invasion by upregulating MT1-MMP expression. Oncogene.

[51] Shi J, Son M-Y, Yamada S, Szabova L, Kahan S, Chrysovergis K (2008). Membrane-type MMPs enable extracellular matrix permissiveness and mesenchymal cell proliferation during embryogenesis. Dev Biol.

[52] Itoh Y, Ito N, Nagase H, Evans RD, Bird SA, Seiki M (2006). Cell surface collagenolysis requires homodimerization of the membrane-bound collagenase MT1-MMP. Mol Biol Cell.

[53] Miller M-C, Manning HB, Jain A, Troeberg L, Dudhia J, Essex D (2009). Membrane type 1 matrix metalloproteinase is a crucial promoter of synovial invasion in human rheumatoid arthritis. Arthritis Rheum.

[54] Huang M, Hayward JJ, Corey E, Garrison SJ, Wagner GR, Krotscheck U (2017). A novel iterative mixed model to remap three complex orthopedic traits in dogs. PLoS One.

[55] Delgado-Calle J, Fernández AF, Sainz J, Zarrabeitia MT, Sañudo C, García-Renedo R (2013). Genome-wide profiling of bone reveals differentially methylated regions in osteoporosis and osteoarthritis. Arthritis Rheum.

[56] Fei Q, Lin J, Meng H, Wang B, Yang Y, Wang Q (2016). Identification of upstream regulators for synovial expression signature genes in osteoarthritis. Jt Bone Spine.

[57] Ahola-Olli AV, Würtz P, Havulinna AS, Aalto K, Pitkänen N, Lehtimäki T (2017). Genome-wide association study identifies 27 loci influencing concentrations of circulating cytokines and growth factors. Am J Hum Genet.

[58] GWAS Catalog. https://www.ebi.ac.uk/gwas/variants/rs1452928. Accessed 18 Jan 2019.

[59] Koch AE, Kunkel SL, Shah MR, Fu R, Mazarakis DD, Haines GK (1995). Macrophage inflammatory protein-Iβ: a C-C chemokine in osteoarthritis. Clin Immunol Immunopathol.

[60] SLC7A1. https://www.uniprot.org/uniprot/P30825#function. Accessed 28 Jan 2019.

[61] Pascale V, Pascale W, Lavanga V, Sansone V, Ferrario P, De Gennaro Colonna V (2013). L-arginine, asymmetric dimethylarginine, and symmetric dimethylarginine in plasma and synovial fluid of patients with knee osteoarthritis. Med Sci Monit.

[62] Boyle EA, Li YI, Pritchard JK (2017). An expanded view of complex traits: from polygenic to Omnigenic. Cell.

[63] Hip joint statistics - German shepherd. In: Breeding database of the Finnish Kennel Club; 2017. https://jalostus.kennelliitto.fi/frmTerveystilastot.aspx?R=166&Lang=en. Accessed 18 Sep 2017.

[64] Flückiger M, Ecvdi D (2008). Scoring radiographs for canine hip dysplasia-the big three organisations in the world. Eur J Companion Anim Pract.

[65] Lust G, Todhunter RJ, Erb HN, Dykes NL, Williams AJ, Burton-Wurster NI (2001). Repeatability of dorsolateral subluxation scores in dogs and correlation with macroscopic appearance of hip osteoarthritis. Am J Vet Res.

[66] R Core Team. R: The R Project for Statistical Computing. R Foundation for statistical computing. 2017. https://www.r-project.org/.

[67] Fraley C, Raftery AE, Murphy TB, Scrucca L. mclust Version 4 for R: Normal Mixture Modeling for Model-Based Clustering, Classification, and Density Estimation. In: Technical Report 597. Seattle: University of Washington; 2012. p. 1–50. http://www.stat.washington.edu/research/reports/2012/tr597.pdf.

[68] Purcell S, Neale B, Todd-Brown K, Thomas L, Ferreira MAR, Bender D (2007). PLINK: a tool set for whole-genome association and population-based linkage analyses. Am J Hum Genet.

[69] Aulchenko YS, Ripke S, Isaacs A, van Duijn CM (2007). GenABEL: an R library for genome-wide association analysis. Bioinformatics..

[70] CanFam3.1 GCA_000002285.2. https://www.ncbi.nlm.nih.gov/assembly/GCF_000002285.3/. Accessed 26 Apr 2018.

[71] Chen W-M, Abecasis GR (2007). Family-based association tests for Genomewide association scans. Am J Hum Genet.

[72] lm {stats} R Documentation. https://stat.ethz.ch/R-manual/R-devel/library/stats/html/lm.html. Accessed 3 May 2019.

[73] glm {stats} R documentation. https://stat.ethz.ch/R-manual/R-devel/library/stats/html/glm.html. Accessed 3 May 2019.

[74] r2fast. https://www.rdocumentation.org/packages/GenABEL/versions/1.8-0/topics/r2fast. Accessed 3 May 2019.

[75] Johnson RC, Nelson GW, Troyer JL, Lautenberger JA, Kessing BD, Winkler CA (2010). Accounting for multiple comparisons in a genome-wide association study (GWAS). BMC Genomics.

[76] Gao X, Becker LC, Becker DM, Starmer JD, Province MA (2010). Avoiding the high Bonferroni penalty in genome-wide association studies. Genet Epidemiol.

