# Supplementary figures and images for: Genetic dissection of canine hip dysplasia phenotypes and osteoarthritis reveals three novel loci
Source: BMC Genomics. 2019 Dec 27;20:1027. doi: 10.1186/s12864-019-6422-6 (PMC6935090; doi:10.1186/s12864-019-6422-6)

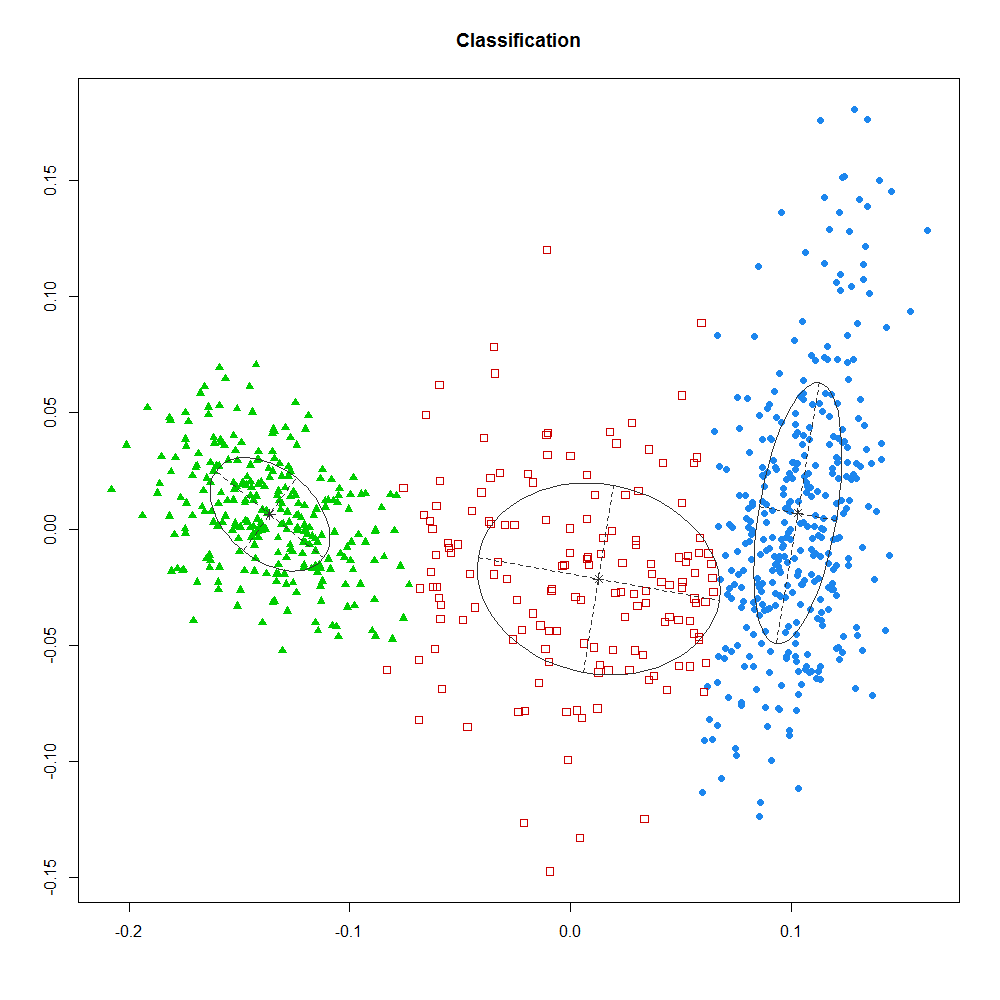

Supplement: Supplementary file 7 — Additional file 7. Classification plot of the population structure from R-package “mclust”. The population is divided into three subpopulations, where the green triangles represent individual dogs of one subpopulation and the red squares and the blue dots represent the individual dogs of the two other subpopulations [file 12864_2019_6422_MOESM7_ESM.tiff]

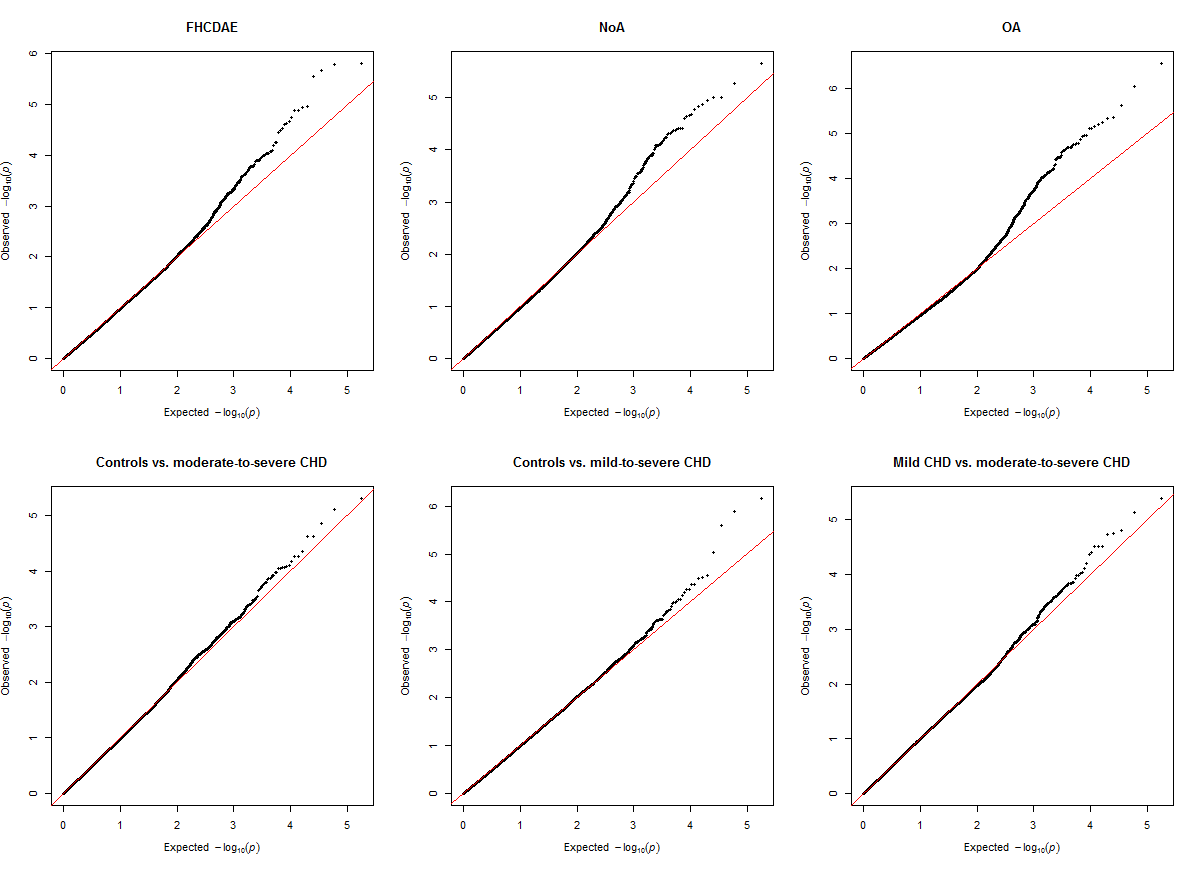

Supplement: Supplementary file 8 — Additional file 8. Q-Q plots of the various association analyses corresponding to Tables 1-4 [file 12864_2019_6422_MOESM8_ESM.tiff]
